# Parkinson’s disease: a never ending story

**DOI:** 10.1007/s00702-023-02631-8

**Published:** 2023-05-04

**Authors:** Heinz Reichmann, Wolfgang Jost

**Affiliations:** University Hospital Carl Gustav Carus, Dresden, Saxony Germany

Once a year, the German Parkinson Expert Group is gathering to discuss the most recent developments and controversies in Parkinson’s disease (PD). Each year, we have the honor of welcoming leading experts from abroad. The format consists of plenary lectures and workshops, so that each participant is fully engaged, and encouraged to contribute to publications. We are very grateful that we have the opportunity to publish our work in the Journal of Neural Transmission. In this issue, the variety of topics, which were covered during our meeting in November 2022, are presented.

It is amazing what has been achieved in the 25 years since our first meeting, in terms of our knowledge of the pathogenesis, diagnosis and available therapies for PD. While we knew some 25 years ago that PD’s motor symptoms result from a dopaminergic deficit in the nigrostriatal pathway, we have extended our knowledge of causative mechanisms to include malformation of alpha-synuclein, mitochondrial damage with disturbances in brain metabolism, and immunologic defects, which may result in cell degeneration, and misfolding of physiological alpha-synuclein. When Braak published his observation that PD is a spreading disease involving the spread of abnormal alpha-synuclein over the brain and its occurrence in the enteric nervous system as well, it was clear that some patients did not fit into this staging. *Per Borghammer* now has an intriguing new (additional) hypothesis, which he calls the connectome theory, and which explains nicely why some patients present with a symmetric form of PD’s motor symptoms and others with an asymmetric form. In addition, his theory also explains why some patients have more non-motor symptoms compared to other patients.

Neurofilament light chain may be a very promising and still novel biomarker, which not only correlates with the stage of PD but also with severity and progression. Choe gives a detailed description of our present knowledge in this field in this issue.

Ever since Polymeropoulos and Golbe not only described a large kindred with a monogenetic defect of alpha-synuclein, but also elucidated the underlying gene defect in detail, the genetics of PD has proven to be a miracle story. This was the start of an enormous input of genetics on our knowledge of PD pathogenesis. It is amazing that starting with this original work on alpha-synuclein, step by step, we have learnt that this protein, when misfolded, is the most important trigger for PD. During the last decades, we have elucidated more and more gene defects, some of which are autosomal dominant (alpha-synuclein, LRRK2) and some of which are autosomal recessive (PINK1, DJ1, Parkin) and cause a gain or loss of function. *Gasser* discusses in his work whether we are at the advent of incorporating gene diagnostics into our regular workup of PD patients.

In our view, wearables may help us, in many ways, to improve the diagnosis and treatment of PD. Since we deal with elderly people, many of whom live far away from medical centers, digital methods such as telemedicine and wearable sensors or apps may help to better diagnose PD, bradykinesia and hyperkinesia. *Reichmann* describes the most recent developments in this field and highlights that continuous registration of movements may be at least equal to registration of mobility in the Hauser diary and that modern apps may warn you, when the voice is analyzed or tapping of letters is changed, that you may be a de novo PD patients.

*Woitalla *et al*.* elegantly discuss the status of dopamine agonists for treatment in PD. Specifically, they claim that dopamine agonists show a rather different dopamine receptor affinity profile, and thus open windows of opportunity to address specific symptoms in patients with PD. In contrast to levodopa, they cause less motor complications but are associated with more side effects.

*Wüllner *et al*.* propose that different triggers for the establishment of so-called idiopathic Parkinson syndrome should be addressed by specific therapy. Thus, inflammation in the brain or gut, oxidative stress and protein misfolding may occur at specific times during the development and propagation of the disease and there may well be different therapeutic windows to counteract these pathological situations.

A hallmark of our meetings is workshops in which new trends and open questions are discussed and as a viewpoint established. *Schroeter *et al*.* discuss red flags that point to atypical Parkinson syndrome. Using a modified Delphi panel, they established distinct symptom patterns for each atypical Parkinson syndrome. Nonetheless, they state in their summary that instead of this attempt, strongly discriminating clinical signs were few.

*Tönges *et al*.* discuss a new approach to make the subtyping of PD more reliable. Using the Bochum PD cohort, they identified three PD clusters: early-onset mild type, intermediate type, and late-onset severe type. There was a good distinction between the clusters, except for the occurrence of polyneuropathy and levodopa dose. In contrast, Parkinson’s Disease Questionnaire, Non-motor Symptom Questionnaire, and the MDS-UPDRS Part II were found to be crucial factors for PD subtype differentiation.

*Regensburger *et al*.* compare the clinical benefit of MAO-B and COMT inhibition in PD. They present a comprehensive overview of the existing information on both classes and their benefit to the treatment of PD and provide an overview on ongoing studies with both kind of inhibitors.

A new levodopa formulation is available in Germany. Inhalation of levodopa may be a new tool to overcome problems such as early morning akinesia, end-of-dose wearing off, delayed on or dose failure. As stated by *Jost *et al*.* this new formulation is a most intriguing on demand therapy with fast response.

*Lingor *et al*.* present future therapies for patients with PD. They elegantly show how new drugs arise from an understanding of PD disease mechanisms. In addition, due to the development of anti-alpha-synuclein antibodies, pre-symptomatic treatment becomes more and more important, and for such studies, it has to be established how early PD is defined.

In summary, it is amazing how many new ideas about the pathogenesis and treatment of PD have been established over the years, and that discussion among PD specialists is a rewarding way in which to improve the condition of our patients.

Heinz Reichmann, Dresden, Wolfgang Jost, Wolfach, April 2023.

## Data Availability

Not applicable.

